# Acknowledgment to the Reviewers of *Pathophysiology* in 2022

**DOI:** 10.3390/pathophysiology30010002

**Published:** 2023-01-16

**Authors:** Pathophysiology Editorial Office

**Affiliations:** MDPI AG, St. Alban-Anlage 66, 4052 Basel, Switzerland

High-quality academic publishing is built on rigorous peer review. *Pathophysiology* was able to uphold its high standards for published papers due to the outstanding efforts of our reviewers. Thanks to the efforts of our reviewers in 2022, the median time to first decision was 23 days and the median time to publication was 50 days. Regardless of whether the articles they examined were ultimately published, the editors would like to express their appreciation and thank the following reviewers for the time and dedication that they have shown *Pathophysiology*:


Ahmed S. AliManuel BlesaAmarpreet SabharwalMara Rúbia CelesAmbar Lopez-MacayMaria KomelkovaAnatoly KubyshkinMartina CebovaAndrás PappMary M. SalvatoreAndrea SambriMary SheppardAndrzej PawlikMarzia Di DonatoAngelos SikalidisMasahiro SugimotoAnna BaraniakMetehan ImamogluAntiño R. AllenMichael J. MyersArkaitz MucientesMing YangAyman ElsamanoudyNadia NajafiAyokanmi OreNatsuo UedaBee Kang TanNicolai V. BovinBernhard ReimersOlivera Mitrović AjtićBingmei FuÖzlem ÖzmenCarmen ConcertoPankaj TrivediCarsten GründkerPasquale FlorioDaniela Vieira BuchaimPeter L. A. M. VosDarko ModunRafael GalupaDavid ZweikerRajesh ParsanathanDiana Cruz-TopeteRanda EshaqDouglas FraserRavindra DeshpandeEisa Tahmasbpour MarzouniRaymond WangEric F. GrabowskiRitthideach YorsaengEsra PolatRobert L. SahEszter EmriSanthosh RajamaniEvangelia FoukaShangru LyuFederico PerfettoSherien El-DalyFiras AbedSlavica KvolikFrancisco Ferreira-da-SilvaSonia MorettiFrancisco José HidalgoStefania CantoreGausal KhanSuling ZengGediminas CepinskasSuncana Simonic-KocijanGerhard P. PüschelSusana Santos BragaGiovanni Francesco SolitroSylviane MullerGislane Lelis Vilela De OliveiraTadayuki OshimaGwang Hyeon EomTae-Yoon LeeHendri SusantoTamas AranyiHiromasa HasegawaTatsuo KandaHoward A. YoungTe-Huei YehIpsita MohantyThanaphum OsathanonIzabela ZakrockaThomas ZöggJerome TannerTommaso FossaliJerzy ChudekTsukuru UmemuraJohari Yap AbdullahUnzué LeireJorge Carlos-VivasUyen TruongJorge H. LeitãoVíctor M. RiveraJorge SimonVittoria Di MauroJuan Peragón-SánchezVladimir VimbergJuei-tang ChengVuokko LoimarantaJungmi YunWerner KilbKatarzyna NeubauerYekbun AdiguzelKoulla ParpaYicheng LongKrzysztof DolowyYolanda García-MesaKuthati YaswanthYubbu PutriLarisa AnghelYu-Chih ChenLarisa Viktorovna SuturinaZhiguo ZhangLorena OrosticaZoltán PozsonyiMaja Sabol



